# Heterologous expression and purification of recombinant human protoporphyrinogen oxidase IX: A comparative study

**DOI:** 10.1371/journal.pone.0259837

**Published:** 2021-11-18

**Authors:** Zora Novakova, Daria Khuntsaria, Marketa Gresova, Jana Mikesova, Barbora Havlinova, Shivam Shukla, Lucie Kolarova, Katerina Vesela, Pavel Martasek, Cyril Barinka

**Affiliations:** 1 Laboratory of Structural Biology, Institute of Biotechnology of the Czech Academy of Sciences, BIOCEV, Vestec, Czech Republic; 2 First Faculty of Medicine, Charles University in Prague, Prague, Czech Republic; CIC bioGUNE, SPAIN

## Abstract

Human protoporphyrinogen oxidase IX (hPPO) is an oxygen-dependent enzyme catalyzing the penultimate step in the heme biosynthesis pathway. Mutations in the enzyme are linked to variegate porphyria, an autosomal dominant metabolic disease. Here we investigated eukaryotic cells as alternative systems for heterologous expression of hPPO, as the use of a traditional bacterial-based system failed to produce several clinically relevant hPPO variants. Using bacterially-produced hPPO, we first analyzed the impact of N-terminal tags and various detergent on hPPO yield, and specific activity. Next, the established protocol was used to compare hPPO constructs heterologously expressed in mammalian HEK293T17 and insect Hi5 cells with prokaryotic overexpression. By attaching various fusion partners at the N- and C-termini of hPPO we also evaluated the influence of the size and positioning of fusion partners on expression levels, specific activity, and intracellular targeting of hPPO fusions in mammalian cells. Overall, our results suggest that while enzymatically active hPPO can be heterologously produced in eukaryotic systems, the limited availability of the intracellular FAD co-factor likely negatively influences yields of a correctly folded protein making thus the *E*.*coli* a system of choice for recombinant hPPO overproduction. At the same time, PPO overexpression in eukaryotic cells might be preferrable in cases when the effects of post-translational modifications (absent in bacteria) on target protein functions are studied.

## Introduction

Heme serves as a prosthetic group of a variety of proteins involved in fundamental biological processes including photosynthesis, respiration, oxygen transport, and detoxification. Heme biosynthesis is a multi-stage process involving eight enzymatic reactions [1]. Protoporphyrinogen oxidase IX (PPO) is an oxygen-dependent enzyme catalyzing the penultimate step in the heme biosynthesis, converting protoporphyrinogen IX to protoporphyrin IX, a precursor of chlorophylls in plants as well as hemoglobin in animals [2]. Given a critical importance of heme in physiology of living organisms, PPO is a focus of intensive basic and applied research. As an example of the latter, plant PPOs are targeted by a number of herbicides to control weeds to sustain and improve agricultural production [3, 4]. On the other side of the spectrum, human PPO (hPPO) is intensively studied in relation to human well-being, as mutations in this enzyme cause variegate porphyria, an autosomal dominant disease with diverse neurological and cutaneous manifestations. More than 180 mutations of hPPO, including deletions, splice variants and missense mutations have been identified so far. Missense mutations account for over 50% of these hPPO variants and in general reduce specific hPPO activity leading to variegate porphyria [5–9].

Human PPO is synthesized in the cytosol and transferred to the inner mitochondrial membrane [10–14], where it forms a complex with ferrochelatase, the ultimate enzyme of the heme biosynthetic pathway [15–18]. As hPPO does not contain a membrane-spanning region and is only loosely associated with the mitochondrial membrane, it can be solubilized in the active form by the use of mild detergents [2, 19–23]. Detergents are also used during purification of heterologously produced hPPO to mask hydrophobic patches on the protein surface. Structurally, hPPO consists of 3 domains: the membrane-binding domain (residues 92–209), the FAD-binding domain (residues 1–91, 210–310 and 417–477), and the substrate-binding domain (residues 311–416) [9, 24]. It shall be noted that all eukaryotic PPOs contain flavin adenine dinucleotide (FAD), a noncovalently associated cofactor that uses molecular oxygen as the terminal acceptor of electrons and is indispensable for PPO enzymatic activity [22, 23, 25].

hPPO has been cloned from human placenta, heterologously expressed in *E*.*coli*, and purified to homogeneity for the first time by Dailey and coauthors [26]. Since then, this purification protocol (and its slight modifications) comprising *E*.*coli* expression and Ni-NTA affinity purification followed by a size-exclusion chromatography step is typically used in the field to produce hPPO and its variants for *in vitro* biochemical, biophysical and structural studies [24, 26–28]. While prokaryotic expression is quite robust in the case of wild-type hPPO, here we wanted to explore avenues for hPPO large-scale heterologous expression in eukaryotic cells and compare characteristics of hPPO produced in the prokaryotic *vs* eukaryotic system. Additionally, we also report the impact of detergents on the hPPO specific activity as such data are not available in existing publications [24, 29].

## Materials and methods

Unless stated otherwise, all chemicals were purchased from Sigma-Aldrich (St. Louis, MO, USA).

### Cell lines

Suspension-adapted HEK293T17 cells (kindly provided by Ondrej Vanek, Charles University, Prague, Czech Republic) were grown in the Free Style F17 medium (ThermoFisher Scientific, Waltham, MA, USA) supplemented by 0.1% Pluronic F-68 (Invitrogen, ThermoFisher Scientific) and 2 mM L-glutamine at 110 rpm under a humidified 5% CO_2_ atmosphere at 37°C. U-2 OS cells were grown in the D-MEM high glucose medium supplemented with 10% v/v FBS under humidified 5% CO_2_ atmosphere at 37°C. Insect Sf9 and Hi5 cells (provided by Dr. Sacha, IOCB, Prague, Czech Republic) were cultivated in Insect-XPRESS medium (Lonza, Basel, Switzerland) at 27°C by shaking at 100 rpm.

### hPPO sub-cloning

**Fig 1:**
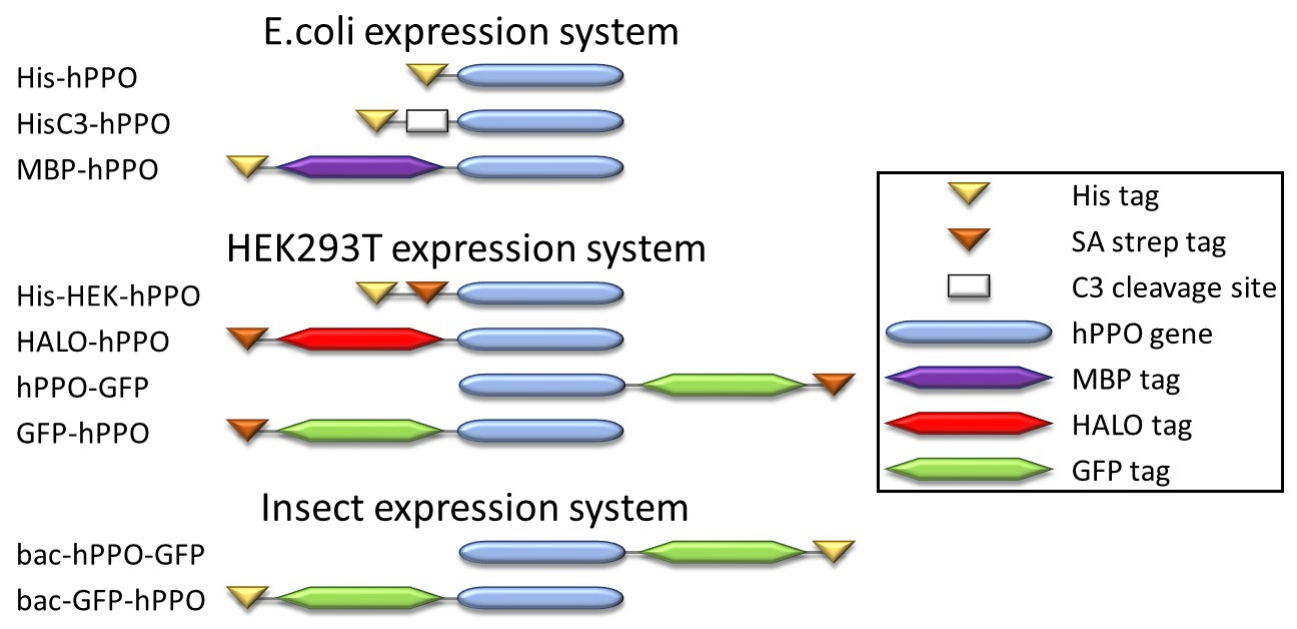
Schematic representation of hPPO constructs used in this study. C3 – C3-protease recognition site; Strep–Strep-tag; His–His tag.

A plasmid for bacterial expression of His-hPPO was kindly provided by Dr. Harry A. Dailey (pTrcHis_B_PPO plasmid; University of Georgia, Athens, GA, USA; [26]). To clone the pHisC3_hPPO plasmid, the C3-protease cleavage site was inserted between the His tag and the hPPO coding sequence in the pTrcHis_B_PPO plasmid using standard PCR. Remaining plasmids were prepared using the Gateway cloning methodology. To this end, we ordered a codon-optimized sequence encoding the hPPO (ThermoFisher) and inserted it into the pDONR221 vector using the BP enzyme mix (ThermoFisher). The identity of the resulting entry clone was confirmed by Sanger sequencing. Expression plasmids were generated via LR recombination reaction between the entry clone and a required Gateway-compatible destination vector. hPPO variants with an N- and C-terminal tag intended for expression in insect cells were prepared by insertion of the hPPO gene into the pOCC65 and pOCC61 vector backbone, respectively [30]. AscI and NotI restriction sites were used for introduction of hPPO gene in vectors. Schematic representations of constructs used in this study are shown in Fig 1.

### hPPO prokaryotic expression and purification

For heterologous expression, E. coli JM109 cells were grown at 30°C as reported previously [26, 31]. Circlegrow media was supplemented with riboflavin to the final concentration of 0.75 μg/mL 2 hours prior to harvesting. Cells were collected by centrifugation (10,000g, 10 min), resuspended in a breaking buffer (50 mM Tris pH 8, 100 mM NaCl, 10% glycerol, 0.5% Tween-20 containing EDTA-free protease inhibitor cocktail (Roche Diagnostics GmbH, Mannheim, Germany), sonicated on ice and centrifuged at 40,000g, 30 min. The supernatant was applied onto a Ni-NTA column (Ni-NTA Superflow, IBA, Germany), washed with 20 column volumes of the equilibration buffer (50 mM Tris-HCl, 100 mM NaCl, 30 mM imidazole, 10% glycerol, 0.02% Tween-20 or 2 mM β-D-glucopyranoside (OGP), pH 8.0) and eluted with the equilibration buffer supplemented with 300 mM imidazole. Elution fractions were pooled and concentrated. The final purification step encompassed size-exclusion chromatography on a Superdex 200 16/60 column (GE Healthcare Life Sciences, Uppsala, Sweden) using 50 mM Tris-HCl, 100 mM NaCl, 5% glycerol, 0.02% Tween-20 (or 2 mM OGP), pH 8.0, as a mobile phase.

### hPPO eukaryotic expression and purification

hPPO constructs were expressed by transient transfection of HEK293T17 cells using linear polyethyleneimine (Polysciences Inc., Warrington,PA, USA) [32]. Three days post-transfection cells were lysed by sonication in an ice-cold lysis buffer (100 mM Tris-HCl, 10 mM NaCl, 5 mM KCl, 2 mM MgCl_2_, 10% glycerol; pH 8.0) supplemented with benzonase (5 U/mL; Merck, Darmstadt, Germany) and the protease inhibitor cocktail (Roche Diagnostics GmbH). Cell lysis was further enhanced by subsequent addition of 0.1% Tween-20 (30 min at 4°C) and 150 mM NaCl for additional 30 min at 4°C. The lysate was cleared by centrifugation at 40000xg for 30 min at 4°C and the recombinant fusion purified by affinity chromatography using StrepTactin XT resin (IBA, Gottingen, Germany). The purified fusion eluted from the column by 10 mM D-biotin (VWR, Radnor, PA, USA) was filtered, flash frozen at concentration 1 mg/mL and stored at -80°C.

Baculoviral expressions in insect cells were done according a published protocol [30]. Briefly, virus particles were prepared by co-transfection of hPPO expression vectors with the DefBac bacmid into Sf9 cells using the Escort IV transfection reagent. P1 virus particles were amplified and used for infection of Hi5 cells. Three days post-infection, cells were lysed by the same procedure as described for HEK293T17 cells. Constructs were purified by affinity chromatography using Ni-NTA resin, eluted by a step gradient of imidazole, filtered and flash-frozen.

### Activity assay

hPPO activity was determined using a fluorescence-based assay quantifying an increase in a fluorescent signal upon conversion of non-fluorescent protoporphyrinogen IX to fluorescent protoporphyrin IX as described previously [31, 33, 34]. Briefly, hPPO was preincubated in a 384-well plate in the total volume of 14 μL for 10 min at 37°C in a reaction buffer comprising 100 mM KH_2_HPO_4_, 0.3% (w/v) Tween-80, 5 mM DTT, 1 mM EDTA, pH 7.2. Reactions were started by the addition of 7 μL of 10 μM protoporphyrinogen IX into the PPO/inhibitor mixture. The fluorescence signal of protoporphyrin IX was monitored with a CLARIOstar fluorimeter (BMG Labtech GmbH, Ortenberg, Germany) at λ_EX_/λ_EM_ = 410/632 nm using a continuous readout mode for 1 hour at 37°C. The reaction velocity was calculated from the linear portion of the measured signal against a standard calibration curve of defined protoporphyrin IX concentrations.

### hPPO cellular localization (immunofluorescence)

U-2 OS cells were passaged onto glass cover slips at the concentration of 35000 cells/mL. The next day, cells were transfected by PPO constructs using the JetPRIME transfection reagent (Polyplus-transfection, Illkirch, France) and cultured for additional two days to allow for the expression of target proteins. For live cell imaging, transfected cells were treated with 50 nM Mitotracker Deep Red FM (ThermoFisher Scientific) for 15 min at 37°C and then by Hoechst 33258 (10 μg/mL) for 30 min at 37°C. Cells were washed and FluoroBrite DMEM medium was added (ThermoFisher Scientific). Coverslips were scanned by a confocal microscope (DMI8, Leica Microsystems, Wetzlar, Germany) equipped with a water immersion objective with magnification 63x and an imaging chamber (OKOlab, Puzzuoli, Italy) maintaining a sample in high humidity and 5% CO_2_ atmosphere at 37°C. In the case of antibody detection, transfected cells were subsequently fixed with 4% formaldehyde for 15 min, permeabilized by 0.1% Triton X-100 for 15 min, washed with PBS and incubated with an anti-PPOX rabbit antibody (Cohesion Biosciences, London, UK) at 4°C overnight. Following the wash with PBS/0.05% Tween-20, slides were treated with a goat anti-rabbit secondary antibody conjugated to Alexa Fluor 594 (Thermo). Slides were stained with 4′,6-diamidino-2-phenylindole (DAPI, 1 μg/mL), washed and mounted in a VectaShield mounting medium (Vector Laboratories, Burlingame, CA, USA). The fluorescence signal was acquired using a DMI8 confocal microscope (Leica Microsystems) equipped with an oil immersion objective with 63x magnification. All images were processed using Adobe Photoshop software (Adobe Systems, San Jose, CA, USA) and analysis of co-localization was done in the ImageJ software (National Institutes of Health, Bethesda, MD, USA).

### SDS PAGE and western blotting

Protein samples were mixed with the Laemmli sample buffer, heated for 5 minutes at 95°C and separated by standard SDS PAGE. Proteins were stained in gel by Coomassie Brilliant Blue G-250 or transferred onto a polyvinylidene difluoride membrane using a semi-dry electroblotting system (Bio-Rad Laboratories, Hercules, CA, USA). The membrane was incubated in a blocking buffer (5% non-fat dried milk/PBS/0.05% Tween-20) for 45 minutes and then treated with an anti-PPOX rabbit antibody (2 μg/mL; Cohesion Biosciences) at 4°C overnight. Goat anti-rabbit secondary antibody conjugated to Alexa Fluor 488 was applied for 1 hour (0.2 μg/mL) and fluorescence signal was visualized using a Typhoon FLA9500 laser scanner (GE Healthcare Life Sciences). Quantity One software (Bio-Rad Laboratories) was used for quantification of the fluorescence signal.

## Results

### N-terminal fusion tags do not influence hPPO enzymatic activity

**Fig 2:**
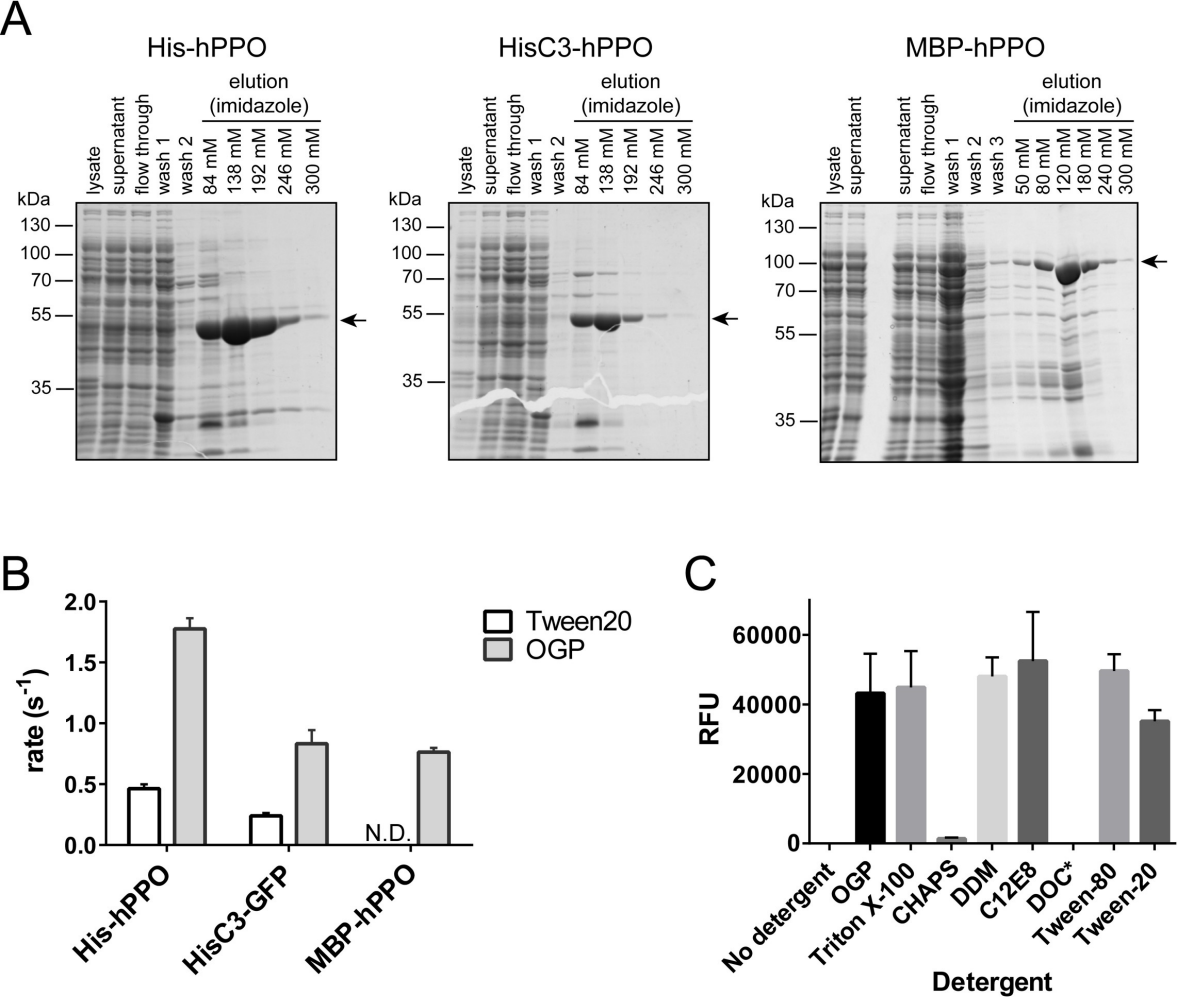
Prokaryotic hPPO expression, purification, and enzymatic activity. **A;** Coomassie-stained SDS-PAGE of hPPO variants purified by Ni-NTA affinity chromatography. Position of constructs marked by arrows. **B;** Substrate conversion rates of *E*.*coli*-expressed hPPO variants using protoporphyrinogen IX as a substrate (N.D., not determined). **C;** Specific activity of hPPO in the presence of various detergents estimated via conversion of protoporphyrinogen IX.

We sub-cloned three variants of hPPO comprising different N-terminal tags: histidine tag (His-hPPO), C3-cleavable His tag (HisC3-hPPO), and His-MBP tag (MBP-hPPO; Fig 1). Corresponding hPPO fusions were expressed in *E*.*coli* and purified to near homogeneity by the combination of Ni-NTA affinity and size-exclusion chromatography (Fig 2A). The overall yields were in the range of 0.6–3.8 mg of the pure protein per liter of the cell culture, with the highest expression observed for His-hPPO (3.8 mg/L). Next, the specific activities of N-terminally tagged proteins were determined using a fluorescence-based assay quantifying conversion of protoporphyrinogen IX to fluorescent protoporphyrin IX. When comparing MBP-hPPO, His-hPPO and HisC3-hPPO (with the N-terminal His tag cleaved), we found less than two-fold differences in the specific activity between the variants purified using the same protocol (Fig 2B and Table 1). These results suggest that the presence of an N-terminal tag does not (negatively) influence the hPPO enzymatic activity and various purification tags can thus be used interchangeably.

**Table 1:** Enzymatic activities of hPPO constructs using protoporphyrinogen IX as a substrate.

Construct	Expression system	Riboflavin (μM)	rate (s^-1^)	Specific activity ratio (%)*
His-hPPO	*E*.*coli*	2	0.5 ± 0.04	26.0
HisC3-hPPO cleaved by C3	*E*.*coli*	2	0.2 ± 0.02	13.5
His-hPPO/OGP	*E*.*coli*	2	1.8 ± 0.09	100.0
HisC3-hPPO/OGP cleaved by C3	*E*.*coli*	2	0.8 ± 0.1	46.9
MBP-hPPO/OGP	*E*.*coli*	2	0.8 ± 0.03	43.0
HALO-hPPO	HEK293T17	10	0.002 ± 0.0004	0.1
HALO-hPPO	HEK293T17	0	0.002 ± 0.0002	0.1
His-HEK-hPPO	HEK293T17	0	N.D.	-
hPPO-GFP	HEK293T17	10	0.02 ± 0.0003	1.2
hPPO-GFP	HEK293T17	0	0.05 ± 0.01	2.8

*specific activity of a given construct compared to the most active enzyme preparation (His-hPPO/OGP)

N.D.–not determined

### Detergent type and concentration have profound influence on hPPO measurable activity

As hPPO is a membrane-associated protein, purification protocols include the use of a detergent additive. Various detergents, including Tween-20, CHAPS, and octyl β-D-glucopyranoside (OGP), have also been used in the past [19, 24, 26, 35, 36]. As Tween-20 forms micelles that are difficult to remove upon size exclusion chromatography and can pose a problem during downstream applications, we used OGP as a primary detergent at 2 mM concentration, which is quite below the critical micellar concentration of 25 mM for this compound. On average, we typically observed >2-fold increase in the total yield of hPPO variants when OGP was included in the purification protocol. Additionally, specific activities of all hPPO variants purified in the presence of OGP were up to 4-fold higher compared to matching variants purified in the presence of Tween-20 (Fig 2B and Table 1). So in our hands, OGP is better suited for purification of highly active hPPO species.

In addition to purification protocols, detergents are also critical components of the PPO assay buffer due to the limited solubility of the protoporhyrin IX reaction product. We thus compared assay buffers comprising different commonly used detergents to find the best performing solution. As shown in Fig 2C, no activity was observed in a control assay buffer without detergent. The use of 0.5% (w/w) deoxycholate (DOC) resulted in solidification of the reaction mixture, while use of lower 0.05% DOC concentration resulted in the absence of any measurable fluorescence of the protoporphyrin fluorescence product. Additionally, the use of 0.7% CHAPS resulted in very low (<5%) hPPO reaction rates. On the other hand, the use of remaining detergents yielded similar specific hPPO activities (expressed as relative fluorescence units) with 0.1% C12E8 and 0.3% Tween-80 being slightly preferred. Overall, our optimized workflow included the addition of 2 mM OGP in purification buffers, while activity assays were performed in an assay buffer supplemented with 0.3% Tween-80.

### hPPO expressed in eukaryotic cells has low specific activity

While wild-type hPPO variants were expressed in the prokaryotic system with yields in low milligrams per liter of media and high specific activities, we failed to produce several of clinically relevant hPPO mutants using *E*.*coli*-based expression system. In the case of pathological hPPO mutants, it is not clear whether unsuccessful prokaryotic expression stems simply from the inherent inability of a given mutant to fold properly or whether prokaryotes are missing critical components, such as suitable chaperons, that would allow production of presumably less stable hPPO mutants. Consequently, we decided to test production of hPPO variants in mammalian HEK293T17 and insect Hi5 eukaryotic expression systems.

**Fig 3:**
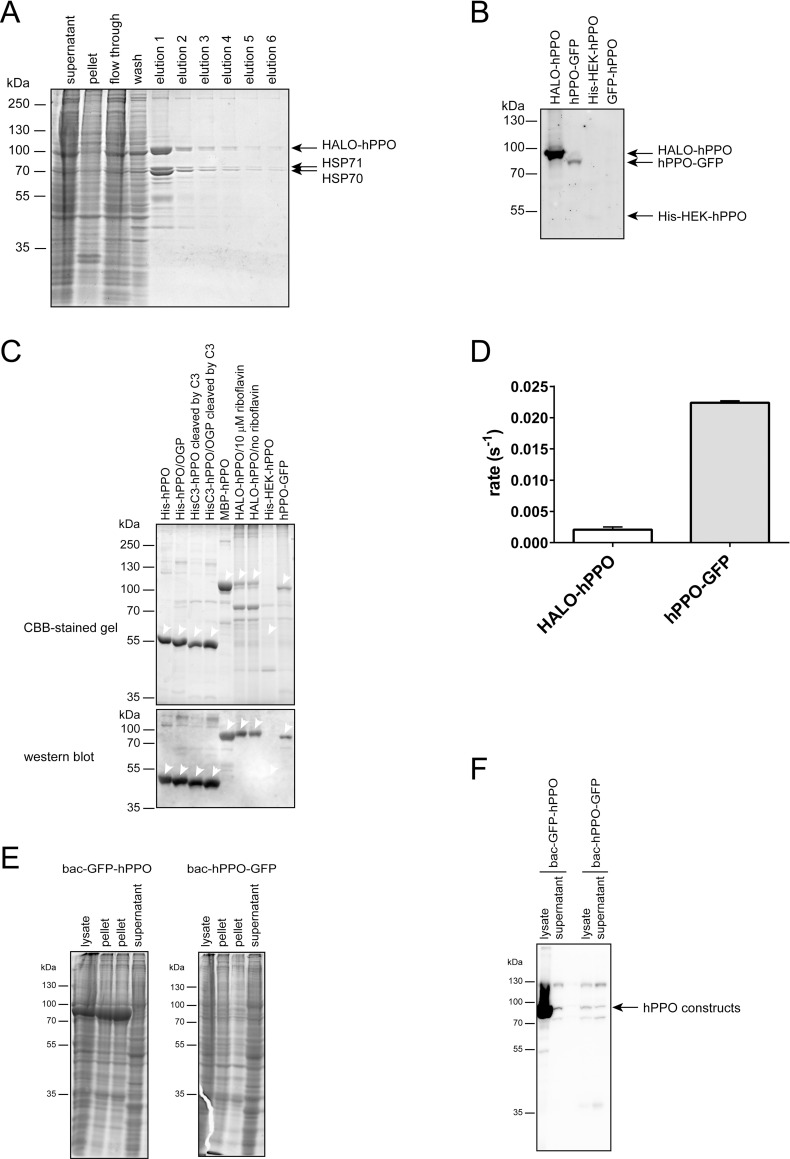
Eukaryotic hPPO expression, purification, and enzymatic activity. **A;** Coomassie-stained SDS-PAGE of HALO-hPPO variant purified by Streptactin affinity chromatography. **B;** Immunodetection of overexpressed hPPO variants in cell lysates by anti-PPOX antibody. **C;** Purified hPPO variants were separated by SDS PAGE and visualized by CBB staining or detected by the anti-PPOX antibody upon electroblotting. Position of each construct marked by an arrowhead. **D;** Specific activities of individual hPPO constructs using protoporphyrinogen IX as a substrate. **E**; Coomassie-stained SDS-PAGE of bac-GFP-hPPO and bac-hPPO-GFP constructs purified by Ni-NTA affinity chromatography. **F;** Immunodetection of overexpressed hPPO variants in cell lysates and supernatants by anti-PPOX antibody.

For hPPO overexpression in HEK293T17 cells, we cloned two hPPO variants with a short His-Strep tag (His-HEK-hPPO) and a substantially bigger Strep-HALO tag (HALO-hPPO; Fig 1) [37]. The latter tag was used as in our experience it markedly increases expression yields of client proteins. Suspension-adapted HEK293T17 cells were transiently transfected using linear polyethyleneimine, harvested three days later, and recombinant fusions purified by Streptactin affinity chromatography (Fig 3A). Overall, the expression yield of the HALO-hPPO fusion was approximately 1 mg/L of cell culture, which is comparable to *E*.*coli* yields. At the same time, expression of the His-HEK-hPPO construct was observable only by microscopic techniques, and we failed to detect overexpressed construct in the cell lysate even by Western blotting (Fig 3B). Furthermore, HEK293T17-expressed hPPO constructs were of limited purity, compared to hPPO variants expressed in *E*.*coli* (Fig 3C). Several contaminating bands were observed in the case of HALO-hPPO. These were later identified by mass spectrometry as heat shock proteins HSP70 and HSP71 (Fig 3A). As the heat shock proteins are typically associated with folding protein intermediates, the presence of HSP chaperones suggests problems with hPPO folding in HEK293T17 cells. The most importantly though, specific activity of HALO-hPPO was 0.002 ± 0.0004 s^-1^, which is more than 100-fold lower compared to bacterially-expressed variants (Fig 3D and Table 1), while the specific activity of His-HEK-hPPO cannot be estimated due to absence of construct in elution fractions.

To exclude unlikely possibility that the HEK293T17 expression system somehow specifically negatively interferes with hPPO expression we complemented our experiments using the baculovirus expression system to produce two hPPO variants, namely bac-GFP-hPPO and bac-hPPO-GFP (Fig 1) featuring GFP- and His-tags at hPPO N- and C- terminus, respectively. Unfortunately, we were not able to purify any reasonable amounts of hPPO from either of the two constructs (Fig 3E and 3F). While in line with HEK293T17 data we observed massive expression levels of the bac-GFP-hPPO construct that comprises a bulky N-terminal tag, virtually all of the fusion protein was present in an insoluble form within cells. At the same time, expression levels of bac-hPPO-GFP were negligible and visible only by Western blotting and we could not detect any hPPO-specific activity in the elution fraction from the StrepTactin column (Fig 3F; Table 1). Overall, given the lower yield, purity, specific activity, and higher production costs of hPPO in eukaryotic cells, our data suggest that eukaryotic expression systems are apparently ill suited for production of recombinant hPPO.

### Effects of FAD on hPPO expression and activity in HEK293T17 cells

**Fig 4:**
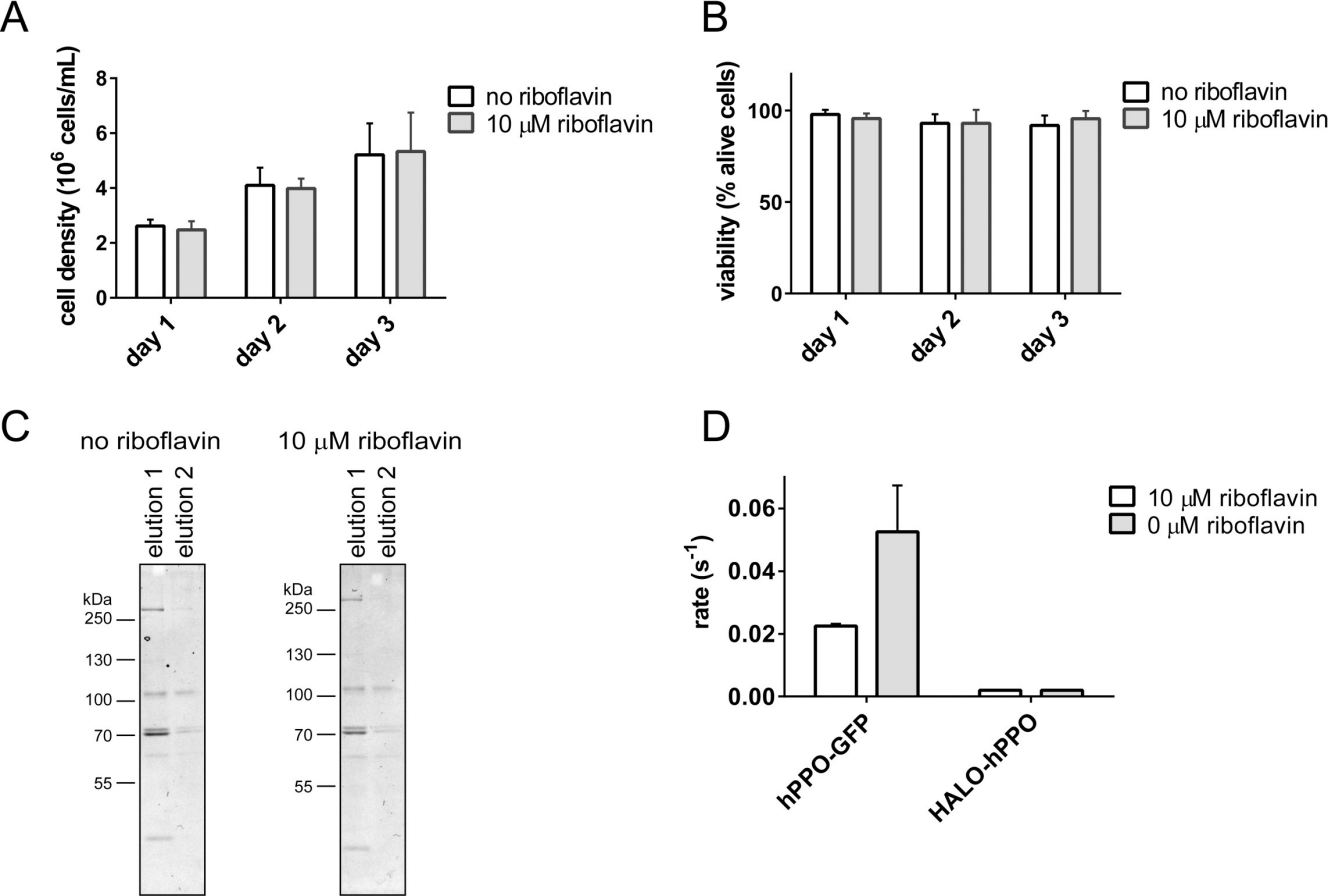
Effect of riboflavin on cell proliferation and viability, and on expression and enzymatic activity of hPPO. **A;** Dependence of cell proliferation on riboflavin media supplementation. Cells were grown in media with or without riboflavin supplementation. **B;** Cell viability in medium with or without riboflavin was determined by Trypan blue assay. **C;** The expression yield of HALO-hPPO in the presence/absence of riboflavin in cultivation media. Fractions eluted from Streptactin affinity column were visualized by Coomassie-stained SDS-PAGE. **D**; The influence of media supplementation with riboflavin on enzymatic activity of hPPO constructs expressed by HEK293T17 cells.

It is not clear, why HEK293T17-produced hPPO has much lower specific activity compared to bacterially expressed proteins. One of the reasons might be limited amounts of intracellular FAD, an hPPO cofactor indispensable for correct hPPO folding and enzymatic activity. During prokaryotic expression of hPPO, the growth medium is typically supplemented with 2 μM riboflavin to optimize hPPO production [26, 38]. In a matching strategy, we repeated expression of HALO-hPPO in media supplemented with 0.01–10 μM riboflavin. The riboflavin supplementation did not have any effect on the growth rate and viability of HEK293T17 cells (10 μM riboflavin-treated cells shown in Fig 4A and 4B) or expression yields (Fig 4C). At the same time, the specific enzymatic activity of HALO-hPPO produced in the presence of 10 μM riboflavin was almost identical compared to the specific activity of HALO-hPPO from non-supplemented media (Fig 4D). Concomitantly, another hPPO construct (hPPO-GFP; described below) expressed in HEK293T17 cells did not show increase in specific activity when supplemented by 10 μM riboflavin (Fig 4C and Table 1). Clearly, contrary to *E*.*coli* heterologous expression, riboflavin supplementation in HEK293T17 cells does not significantly increase the specific activity of hPPO, although the addition of riboflavin to *E*.*coli* culture has superior effect on the activity of overexpressed hPPO constructs.

### Subcellular targeting of hPPO in U-2 OS cells is dependent on the position and size of the fusion tag

Wild type hPPO resides on the matrix side of the inner mitochondrial membrane and mitochondrial targeting can be in principle critical for hPPO enzymatic activity [14, 18]. To investigate this issue in more detail, we constructed a hPPO variant C-terminally fused to GFP (hPPO-GFP), as well as a N-terminal GFP-hPPO and HALO-hPPO fusions (Fig 1). These constructs were used in a series of immunofluorescence and live-cell imaging experiments to determine their intracellular localization in U-2 OS cells.

**Fig 5:**
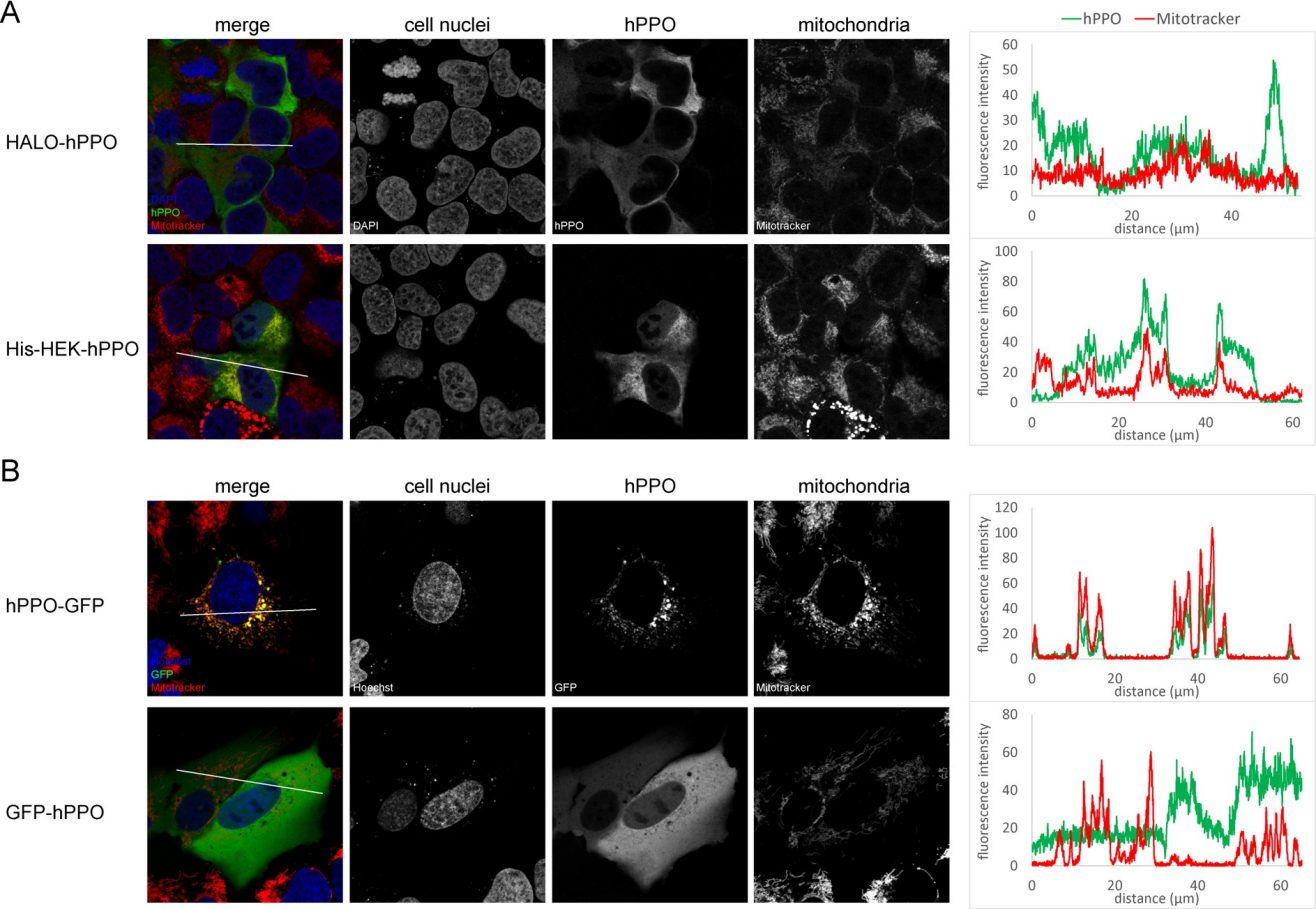
Localization of hPPO constructs in cell. **A;** hPPO variants were transfected into in U-2 OS cells and visualized using PPOX-specific antibody (green channel) with a confocal microscope. Mitochondria were stained by Mitotracker Deep Red FM dye (red channel), whereas cell nuclei were visualized by DAPI staining (blue channel). **B;** Localization of GFP-tagged constructs was detected in live cells by a confocal microscope (green channel). Mitochondria were visualized by Mitotracker Deep Red FM (red channel), whereas cell nuclei were stained by Hoechst 33258 (blue channel). Charts on the right side show the colocalization of hPPO constructs and mitochondria marker Mitotracker in the area marked by a white line.

While HALO-hPPO and GFP-hPPO constructs localized preferentially to the cytosol, His-HEK-hPPO construct was found both in the cytosol as well as in mitochondria labeled with Mitotracker, a mitochondria-specific dye (Fig 5A). Clearly, N-terminal tags prevented, at least partially, translocation of hPPO fusions into mitochondria, with bigger tags being more effective in this respect. To the contrary, the C-terminal hPPO fusion with the GFP tag was localized to mitochondria as evident from the colocalization with Mitotracker (Fig 5B).

While targeting hPPO to mitochondria might decrease the yield of the recombinant protein, it could at the same time have a positive effect on hPPO specific activity. To test this hypothesis, we heterologously expressed the hPPO-GFP construct in HEK293T17 cells in medium supplemented by 15 μM riboflavin. The construct was then purified via StrepTactin affinity chromatography and its specific activity determined. The hPPO-GFP expression yield was approximately 0.2 mg/L, i.e. 5-fold lower compared to HALO-hPPO was significantly lower in cell lysate than amount of HALO-hPPO (Fig 3B). At the same time, surprisingly, the specific activity of hPPO-GFP was 0.022 ± 0.0003 s^-1^, which is 11-fold higher compared to HALO-hPPO, yet still up to 21-fold lower than specific activities of fusions expressed in *E*.*coli*. Apparently, targeting hPPO to mitochondria increases a portion of correctly folded and enzymatically competent hPPO in our preparation, yet with low specific activity compared to the bacterially expressed protein.

## Discussion

Heterologous expression of PPO for biochemical/biophysical studies is typically carried out in *E*.*coli* and virtually no data exist on the use of eukaryotic systems for this purpose, although the small-scale expression of FLAG-tagged enzymes of the heme synthesis pathway in murine MEL cells have been reported recently [18]. Heterologous expression/purification from eukaryotic cells can be beneficial for example in cases when the effects of post-translational modifications (absent in bacteria) on target protein functions are studied. The low specific activity of hPPO produced in the HEK293T17 cytosol is somewhat puzzling as virtually identical constructs produced in *E*.*coli* are much more active. As riboflavin, present in the cell as either flavin adenine dinucleotide (FAD) and flavin mononucleotide (FMN), is an essential cofactor of many redox enzymes including hPPO, we hypothesize that limited availability of riboflavin might be responsible for observed low hPPO specific activity. Unfortunately, the media supplementation with riboflavin did not result in any marked increase of hPPO yields or its specific activity. Mammalian cells are not capable of the de novo synthesis of riboflavin so it must be absorbed from the diet by epithelial cells of small intestine (reviewed by [39]). Riboflavin transport to mammalian cells is mediated by the ubiquitously expressed riboflavin transporter RFT2 with the Km values of 0.21 μM and 0.77 μM for rat and human RFT2, respectively [40, 41]. As a result, the amount of riboflavin in mammalian cells is limited by kinetics of the riboflavin uptake from culture media and its intracellular concentration might not support high demand during overexpression of FAD-dependent hPPO. In contrast to mammalian cells, bacteria have both a riboflavin transmembrane import system (YpaA protein in *E*.*coli*) and also an endogenous riboflavin biosynthesis pathway (reviewed by [42, 43]. Of note, the BL21 *E*.*coli* strain frequently used for heterologous protein expression is capable of the riboflavin synthesis in the range of tens of milligrams per liter of the cell culture [44]. Consequently, given the superior availability of riboflavin in bacterial cells, this system is apparently better suited for expression of FAD/FMN-dependent enzymes compared to mammalian cells.

Overall, while hPPO can be heterologously expressed in eukaryotic cells at reasonable yields, *E*.*coli* are definitely better suited for production of large quantities of highly active hPPO preparations, not only because of lower production costs but mostly because of much higher specific activities of purified enzymes. We reason that the absence of sufficient concentrations of intracellular FAD/FMN is detrimental for hPPO overexpression in eukaryotic cells and these findings shall be considered when selecting a suitable expression host for other flavin-dependent proteins.

## Supporting information

S1 Raw images Raw images of western blots and gels. (PDF)

## Abbreviations

hPPO: human protoporphyrinogen oxidase IX
OGP: β-D-glucopyranoside
MBP: maltose-binding protein
FAD: flavin adenine dinucleotide
FMN: flavin mononucleotide
RFT2: riboflavin transporter 2
