# Supplementary material for: Heterologous expression and purification of recombinant human protoporphyrinogen oxidase IX: A comparative study
Source: PLoS One. 2021 Nov 18;16(11):e0259837. doi: 10.1371/journal.pone.0259837 (PMC8601502; doi:10.1371/journal.pone.0259837)

Supplementary figure 1 - raw images of western blots and gels

Raw image 2A - CBB-stained gel

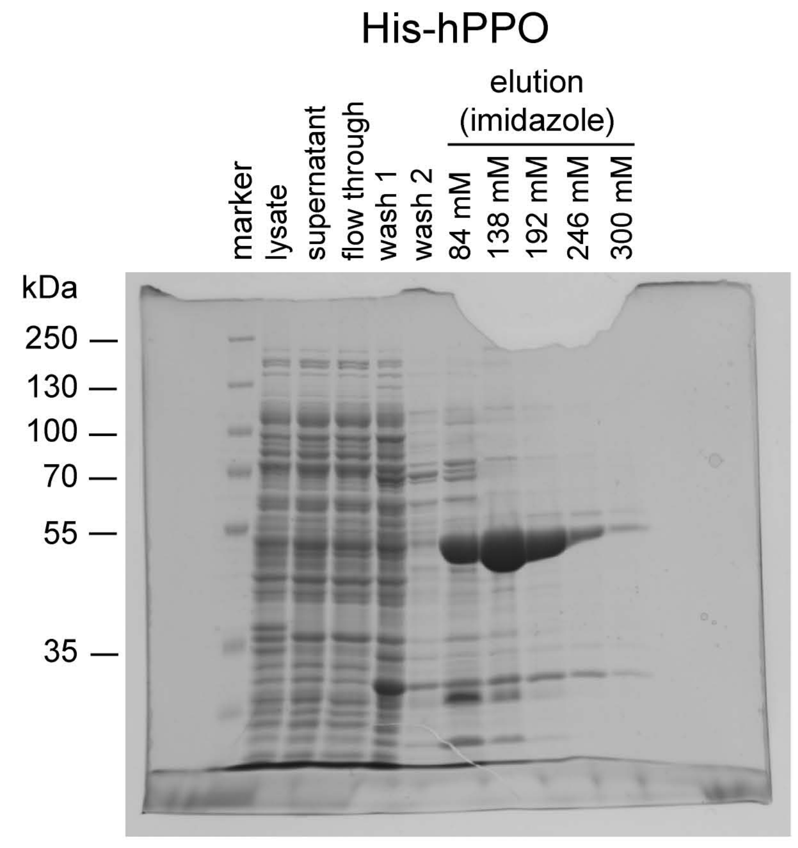

Raw image 2B - CBB-stained gel

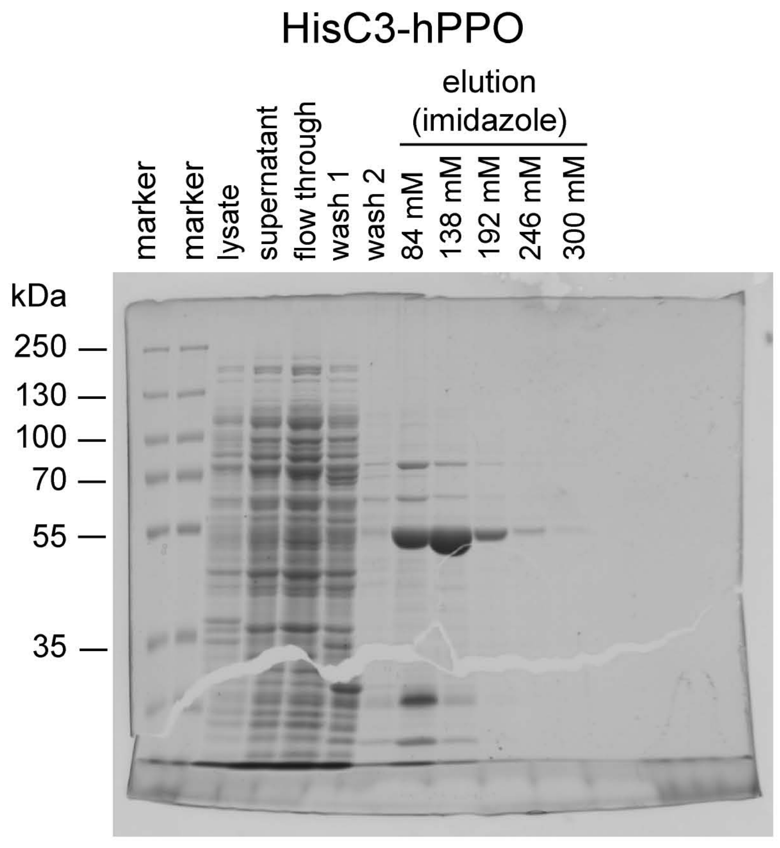

Raw image 2C - CBB-stained gel

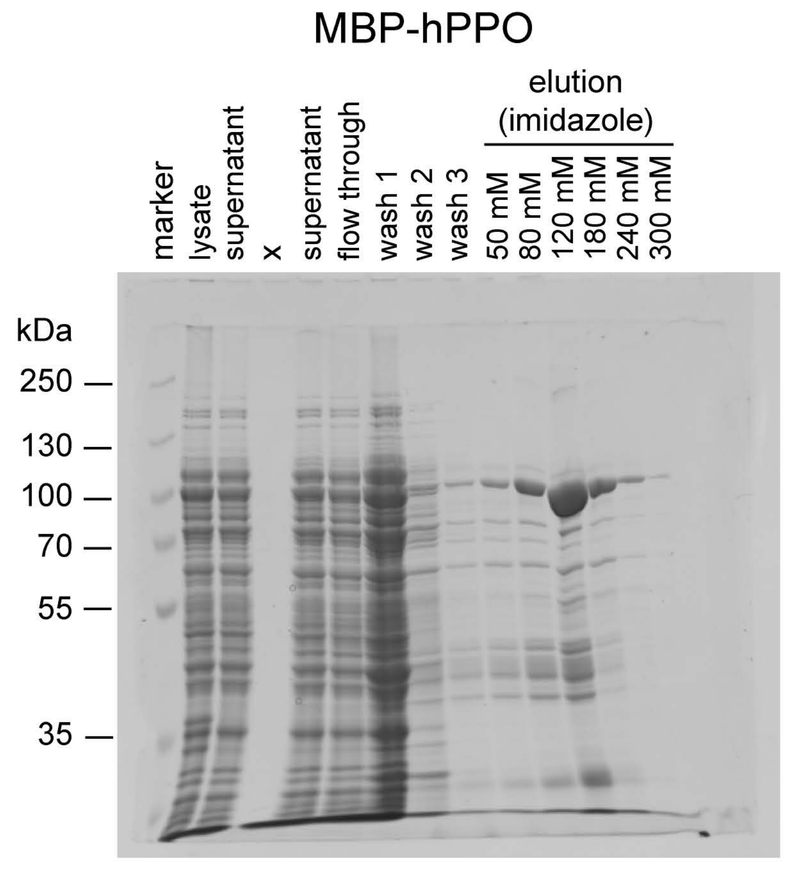

Raw image 3A - CBB-stained gel

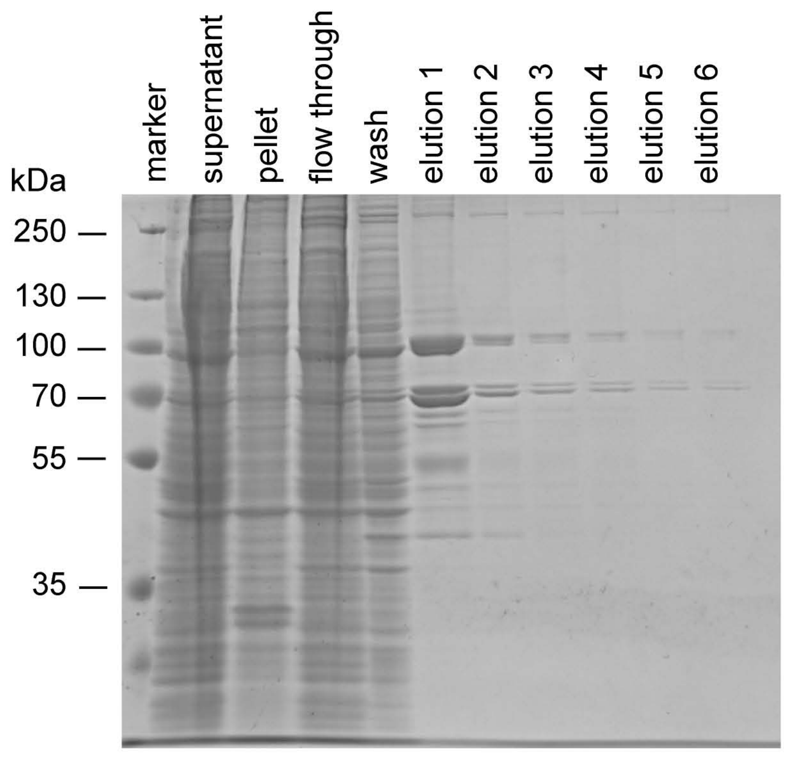

Raw image 3B - western blot

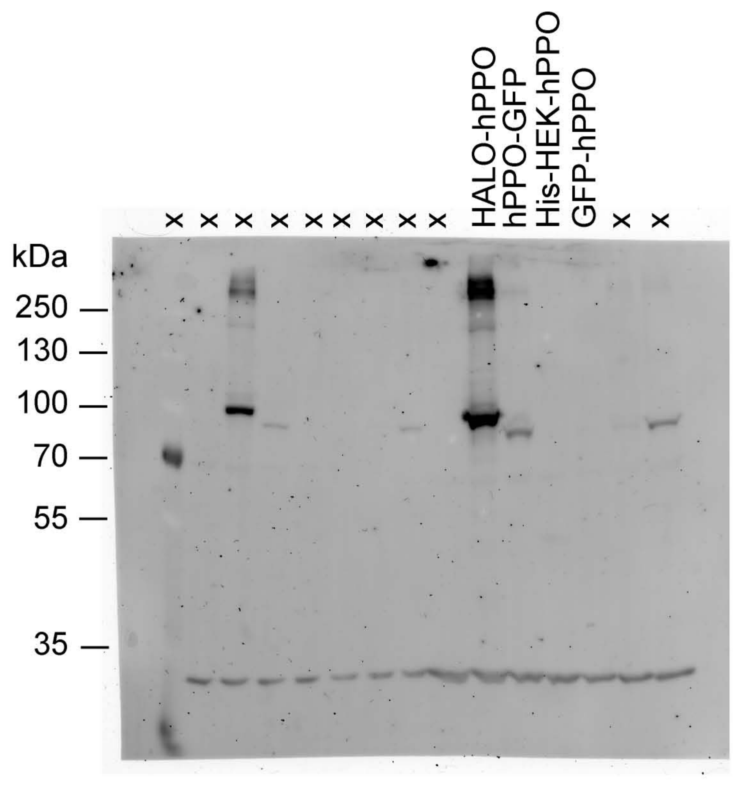

Raw image 3C

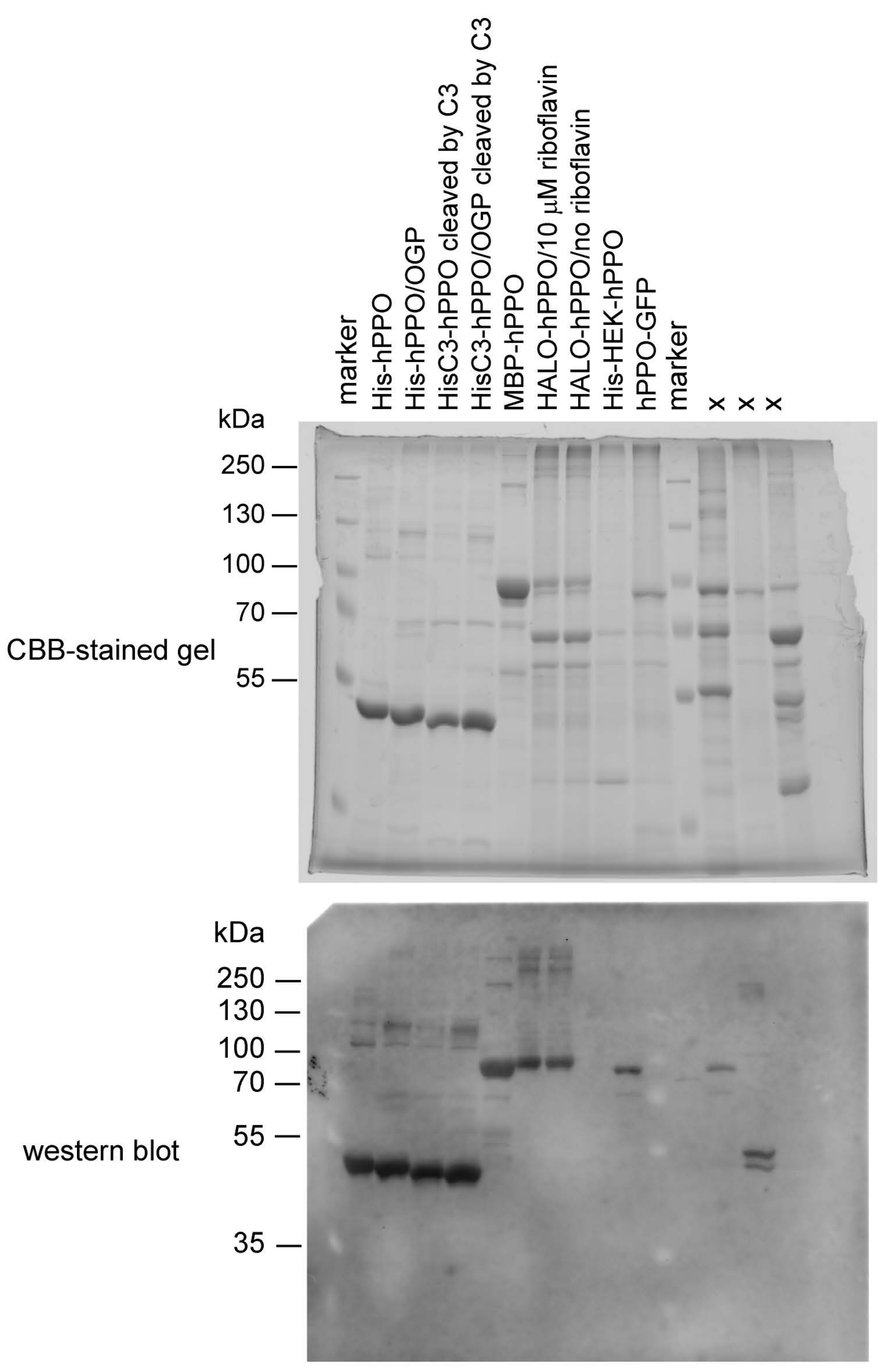

Raw image 3F - western blot

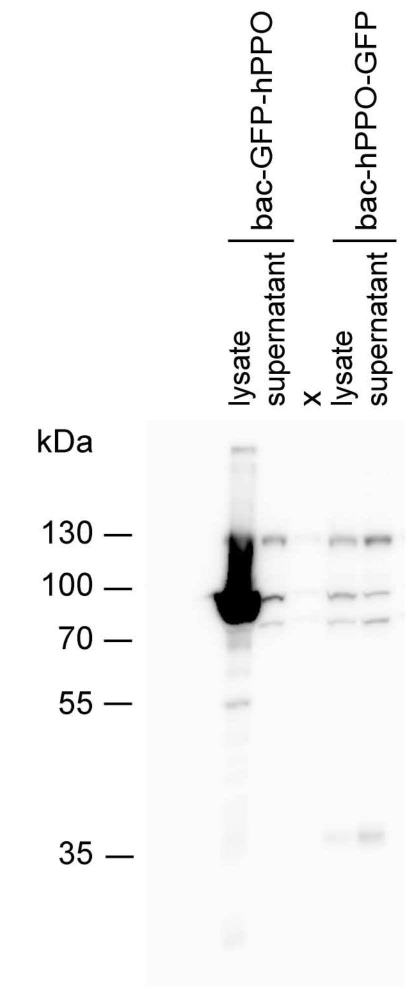

Raw image 3E - CBB-stained gel

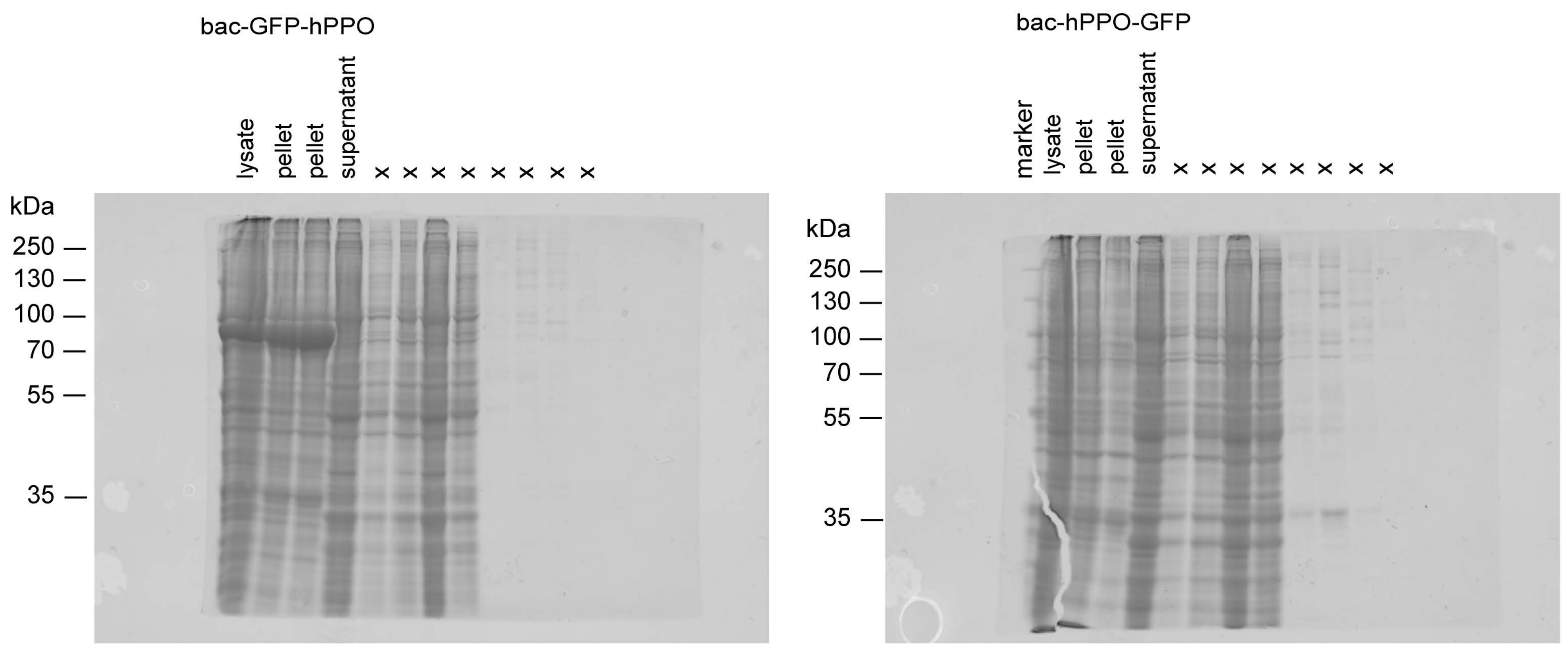

Raw image 4C - CBB-stained gel

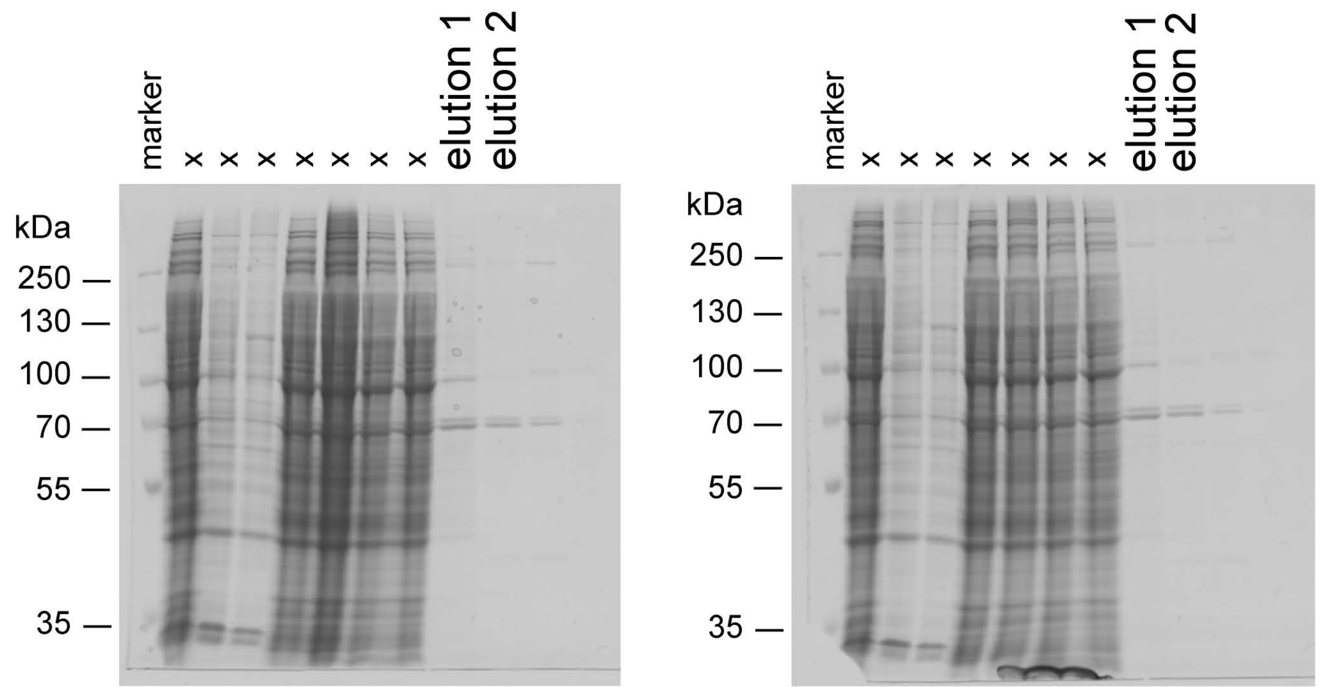

Supplement: S1 Raw images — (PDF) [file pone.0259837.s001.pdf]
